# Yield of summer maize hybrids with different growth duration determined by light and temperature resource use efficiency from silking to physiological maturity stage

**DOI:** 10.3389/fpls.2022.992311

**Published:** 2022-09-29

**Authors:** Jiyu Zhao, Baizhao Ren, Bin Zhao, Peng Liu, Jiwang Zhang

**Affiliations:** State Key Laboratory of Crop Biology and College of Agronomy, Shandong Agricultural University, Tai’an, China

**Keywords:** maize, harvest index (HI), resource use efficiency (RUE), yield, dry matter

## Abstract

In order to explore the physiological mechanism of different yield of summer maize (*Zea mays* L.) hybrids with different growth duration, a field experiment was conducted to study the growth stage, leaf photosynthetic characteristics, dry matter accumulation (DMA), transport and distribution characteristics and yield of the early maturity hybrid Denghai 518 (DH518) and the mid-late maturity hybrid Denghai 605 (DH605) from 2017 to 2021. The results showed that the yield of DH605 was significantly higher than that of DH518. The growth period of DH518 was 7-10 days shorter and the days of the growth stage of the sowing-silking stage (R1) were 5-6 days shorter compared to that of DH605. The contribution to grain dry matter by leaf and stalk dry matter remobilization (DMRC) of DH518 was significantly higher than that of DH605. There was a significant negative correlation between pre-silking growth days and harvest index (HI). The ^13^C distribution to grains of DH518 was significantly higher than that of DH605, and the HI and the corresponding contribution of HI to yield was also higher than that of DH605. The light and temperature resource use efficiency from silking to physiological maturity stage of DH605 was significantly higher than that of DH518. The yield per GDD of DH605 increased by 7.25% than that of DH518. At post-silking, the duration of higher leaf area index (DLAI) (>56 days) and active photosynthesis duration (APD) (>50 days) of DH605 were longer compared with that of DH518, and the average plant growth rate was 7.15% higher than that of DH518, which significantly increased the DMA of DH605. Therefore, the significant reduction of DH518 yield compared with DH605 was not due to the shortening of the growth stage of sowing-R1, but the lower light and temperature resource use efficiency from silking to physiological maturity stage.

## Introduction

Maize has become the largest cereal food crop in China since 2013, which was responsible for 22% of the global maize output (Liu et al., 2017; FAO, 2020). Increasing maize production is critical to ensuring global food and energy security in China (Liu et al., 2020). In a global crisis like COVID-19, food insecurity and disparity can become a serious problem not only in densely populated developing countries (Guha and Chandra, 2021; Odunitan-Wayas et al., 2021) but also in some developed countries (O’Hara and Toussaint, 2021; Oncini, 2021). Under the wheat-maize double-cropping model in the North China Plain, light and heat resources are not enough, so it is impossible to guarantee sufficient maize grain filling duration and late grain dehydration time (Sun et al., 2007; Wang et al., 2012). In order to increase yield, farmers are accustomed to plant mid-late maturity or late maturity hybrids for a long time (Edwards et al., 2005). However, with the wide application of the whole mechanized maize planting mode, the hybrids with a long growth period and slow dehydration in the later stage are increasingly unsuitable for production requirements. Due to the shortening of the growth period, the yield of early maturity hybrids is lower than that of middle-late maturity hybrids (Zhao et al., 2021). Therefore, how to improve the yield of early maturity hybrids becomes an urgent problem to be solved.

Aboveground dry matter accumulation (DMA) and harvest index (HI) are two simple and valuable characteristics for evaluating plant traits and improving yield (Donald and Hamblin, 1976). The characteristics of DMA, distribution, and transfer during the growth period are the important factors to determine the yield of maize (Saidou et al., 2003). The fundamental way to achieve a high yield is to increase DMA and distribution to grains (i.e. HI) as much as possible (Chen, 1994). With the upgrading of hybrids, the DMA per plant also increased, especially at post-silking (Hu et al., 1998). The post-silking DMA accounts for 50% of the total DMA at R6, which is mainly supplied to the grain (Rajcan and Tollenaar, 1999; Tollenaar et al., 2004; Lee and Tollenaar, 2007). In addition, the HI has improved (Echarte and Andrade, 2003; Hou et al., 2012; Ma et al., 2014). However, other studies have shown that the HI does not change continuously over time (Meghji et al., 1984; Tollenaar, 1989). Therefore, it is necessary to explore the differences between DMA and HI and their contribution to the yield of summer maize hybrids with different growth duration.

Leaves are the decisive organs for photosynthesis in terrestrial plants (Piazza et al., 2005). Higher LAI, longer active photosynthesis duration and slower leaf senescence play a positive role in the increase of DMA and yield after silking (Ma and Dwyer, 1998; Borrell et al., 2001; Duvick, 2005). Temperature plays an important role in the growth and development of crops (Lin et al., 2011). It affects the synthesis and distribution of photosynthetic matter in maize (Ptaszynska and Silesia, 2008) and the duration of growth period (Ileleji et al., 2007). In addition, the light and temperature resource use efficiency of high-yield maize hybrids are also higher (Slattery and Ort, 2021). DH605 is one of the most popular maize hybrids in China at present, and DH518 is a newly released hybrid that is being promoted. DH605 (mid-late hybrid) and DH518 (mid-early hybrid) have different growth duration, which are widely representative (Ma et al., 2022). Therefore, we analyzed the yield, accumulated temperature demand characteristics, leaf photosynthetic characteristics, and DMA and distribution characteristics of different summer maize hybrids to explore the effects of the changes in the growth duration on DMA and distribution and yield. We aimed to determine: a) whether the changes in growth period affect the contribution of dry matter and HI to yield, and b) what is the physiological mechanism of yield difference of summer maize hybrids with different growth duration. These results will provide an important reference for improving the yield of early maturity hybrids adapted to mechanical grain harvest.

## Materials and methods

### Plant materials and experimental design

**Figure 1:**
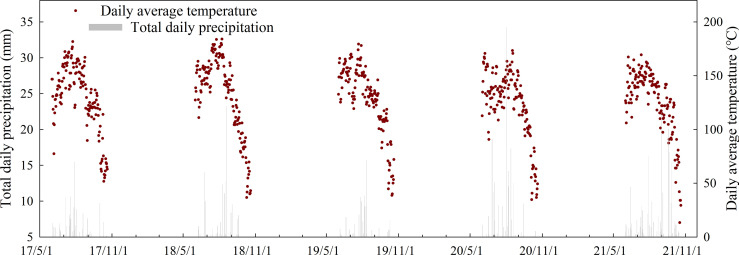
The weather conditions during the summer maize growth duration (2017-2021).

Field experiments were conducted at an experimental farm of Shandong Agricultural University (36.09°N, 117.09°E) from 2017 to 2021. The weather conditions of the summer maize growing season in the planting area are shown in **Figure 1**. The early maturity hybrid Denghai 518 (DH518) and the mid-late maturity hybrid Denghai 605 (DH605) were used as test materials with a row spacing of 60 cm and a planting density of 75,000 ha^-1^. Both hybrids are widely planted in the province of Shandong, China. The hybrid maturity is classified as 113 d for DH605 and 103 d for DH518. The growing season duration (from sowing to harvest dates) for two hybrids in different years were shown in **Table 2**. Each treatment was repeated three times, in a completely randomized design, and the plot area was 54 m^2^ (9 m × 6 m). The N, P, and K fertilizers were applied as base fertilizer: 84 kg ha^-1^ N (urea, 46% N), 52.5 kg ha^-1^ P_2_O_5_ (calcium superphosphate, 17% P_2_O_5_), and 67.5 kg ha^-1^ K_2_O (muriate of potash, 60% K_2_O). At the ninth leaf stage (V9) 126 kg ha^-1^ N fertilizer (urea, 46% N) was applied as supplementary fertilizer.

### Investigation of growth stage and calculation of the growing degree days

After sowing, the date on which the plant reached the sixth leaf stage (V6), the 12th leaf stage (V12), silking stage (R1), milk stage (R3), 45 days after silking (R5) and physiological maturity stage (R6) were observed and recorded. The R6 is defined as the date when the black layer forms at the base of the kernel and the milk line has disappeared. The weather conditions data were provided by the Agricultural Experimental Station of Shandong Agricultural University. GDD was calculated as follows (Gregory and Wilhelm, 1997):


$$[({\text{T}}_{\text{max}}+{\text{T}}_{\text{min}})/2]-{\text{T}}_{\text{base}}$$

where T_max_ is the daily maximum temperature, T_min_ is the daily minimum temperature, T_base_ is the maize growth base temperature (10 °C).

### Leaf area index

Six typical plants were signed from each plot at V6, V12, R1, R3, R5 and R6 stage to measure and calculate leaf area and LAI, according to the method of (Montgomery, 1911).

### Chlorophyll content

Ten typical plants leaves SPAD value was measured as leaf chlorophyll content at V6, V12, VT, R3 and R6 stage. SPAD value of functional leaf was metered using a chlorophyll meter (SPAD-502, Minolta, Japan), and replicated at least eight times (Wu et al., 1998).

### Leaf gas exchange parameters

The net photosynthetic rates (*P_n_*), stomatal conductance (*G_s_*) and intercellular CO_2_ concentration (*C_i_*) were measured in the middle of five ear leaves representational in each treatment from 10:00 to 12:00 at R1, R3 and R5 stage by using a portable infrared gas analyzer (CIRAS-III, PP System, Hansatech, USA). The active photosynthesis duration (APD) and the duration of higher LAI (DLAI) was defined by (Zhang and Cheng, 1992) as the number of days when *P_n_* and LA decrease from the maximum to 50%.


$$Canopy\;photosynthetic\;capacity\;=\;P_{n}\times \;LAI$$

### Accumulation, distribution and transport of dry matter

5 randomly selected plants from each plot were sampled at each physiological stage. At R1 and R6 stage, plants were separated into leaf, stalk (at R1 and R6 stage), and reproductive parts (bract, cob, and grain at R6 stage) for analysis. Plant samples were killed at 105 °C for 30min, then dried at 80 °C to constant weight, and the dry matter weight was measured. Post-silking DMA in aboveground biomass (PoSDMA), Pre-silking and post-silking DMA ratioin aboveground biomass (PrSDMAR and PoSDMAR), dry matter remobilization efficiency (DMRE) of vegetative tissues, and contribution to grain dry matter by leaf and stalk dry matter remobilization (DMRC) were estimated according to the following formulas (Mi et al., 2003; Chen et al., 2014; Chen et al., 2015b):


PrSDMAR(%) =  (Total DMA at R1)/(Total DMA at R6)×100

$$PoSDMA\left(g\;plant^{-1}\right)\;=\text{ Total DMA at R}6-\text{Total DMA at R}1$$

PoSDMAR(%) =  (PoSDMA)/(Total DMA at R6)×100

 DMR of leaf or stalk(g) =  leaf or stalk DMA at R1−leaf or stalk DMA at R6

DMRE(%) of leaf or stalk = (DMR of leaf or stalk/leaf or stalk DMA at R1)×100

$$DMR\;of\;leaf\;or\;stalk\;to\;grain\left(g\right)\;=\text{DMR of leaf or stalk}\times {\;}^{13}\text{C distribution to grains}$$

DMRC(%) of leaf or stalk = (DMR of leaf or stalk to grain/grain DMA at R6)×100

Harvest index was calculated as per (Ciampitti et al., 2013):


HI =  Grain DMA at R6/Total DMA at R6

### ^13^C Pulse labeling, sampling and analysis

At R1 stage, five plants were selected for ^13^CO_2_ labeling and five for reference from the different summer maize hybrids in 2017, 2018, 2020 and 2021. The ear leaf was placed in the labeling chamber (a transparent plastic oven bag sealed at both ends) and 60 mL of ^13^CO_2_ air was pumped into the labeling chamber. After one hour, the bags were removed from the plants. At R6 stage, three labeled ^13^CO_2_ plants and three reference plants without labeled ^13^CO_2_ were separated into stalk, leaf, grain and others. These samples were dried, weighed, and ball-milled for analysis (Schussler and Westgate, 1994).

### Yield and yield components

Thirty ears from the middle three rows of each plot were sampled to measure yield and yield components. Maize kernels with 14% moisture content were determined.


$$Yield\;per\;GDD\;(kg\;ha^{-1}{\left({}^{\circ}C\;d\right)}^{-1})\;=\text{Yield}/\text{Total GDD}$$

; $$Sink\;capacity\;\left(g\;m^{-2}\right)\;=\text{ Number of ears per unit area at R}6\times \text{Grains per ear}\times \text{grain weight}$$

$$Grain/leaf\;\left(kg\;m^{-2}\right)\;=\text{ Sink capacity}/\text{LA at R}1\;stage$$

### Data analysis

Microsoft Excel 2016 (Microsoft, Redmond, WA, USA) and SigmaPlot 12.5 (Systat Software, Inc., Richmond, CA, USA) were used for data processing and plotting, curve expert 1.4 for model fitting. Differences between years or hybrids were compared using ANOVA and Student’s t test with *p*< 0.05 (LSD 0.05). Correlation were carried out according to the method of Piepho (2018). Correlations and regression analyses were estimated as Pearson’s correlation coefficient. The main effects of year, hybrid, and their interactions were tested for the grain yield, dry matter remobilization, DMRC, Grain/leaf and HI using IBM SPSS Statistics 21 (IBM Corporation, Armonk, NY, USA).

## Results

### Yield

There was no significant year × hybrids interaction effects on the yield (**Table 1**). The yield of DH605 was significantly higher and was increased by 3.4-7.3%, compared with that of DH518. The increase in maize yield was mainly due to the significant increase in the 1,000-grain weight of DH605 (**Table 1**). There was no significant difference in the number of ears and grain number per ear (except in 2019) among summer maize hybrids with different growth duration (**Table 1**). The effective accumulated temperature of DH605 after silking was 53.1 °C lower than that of DH518 in 2019 because the sowing date is more than 6 days later than that of other years, resulting in a decrease in 1000-grain weight of DH605 (**Table 2**).

**Table 1:** Grain yield and yield components of summer maize hybrids with different growth duration from 2017 to 2021.

Year	Hybrid	Grains per ear	1000-grain weight(g)	Harvest ear number(10^4^ ha^-1^)	Yield(kg ha^-1^)
2017	DH518	497 ± 5.9a	355 ± 2.5b	6.91 ± 0.06a	12245 ± 145.1b
	DH605	487 ± 6.7a	378 ± 2.2a	6.94 ± 0.05a	12796 ± 130.6a
2018	DH518	495 ± 14.5a	349 ± 1.8b	6.86 ± 0.07a	11860 ± 237.7b
	DH605	514 ± 10.7a	361 ± 1.0a	6.86 ± 0.08a	12720 ± 107.1a
2019	DH518	521 ± 7.0b	359 ± 2.3a	6.57 ± 0.18a	12286 ± 173.5b
	DH605	570 ± 8.7a	334 ± 1.5b	6.67 ± 0.08a	12701 ± 151.8a
2020	DH518	518 ± 12.6a	346 ± 3.4b	6.83 ± 0.09a	12238 ± 214.3b
	DH605	520 ± 10.1a	363 ± 2.0a	6.89 ± 0.17a	13009 ± 289.0a
2021	DH518	543 ± 12.0a	299 ± 2.4b	7.32 ± 0.09a	11889 ± 385.8b
	DH605	561 ± 3.8a	308 ± 2.1a	7.39 ± 0.16a	12754 ± 272.4a
Year (Y)		41.899^**^	734.276^**^	79.543^**^	2.221
Hybrid (H)		18.446^**^	79.723^**^	1.613	71.685^**^
Y × H		7.889^**^	107.430^**^	0.872	1.115

In the same year, means followed by a common letter are not significantly different by LSD test at the 5% level of significance. DH518, Denghai518; DH605, Denghai605. ^**^p< 0.01.

**Table 2:** Growth process and effective accumulated temperature of summer maize hybrids with different growth duration from 2017 to 2021.

Year	Hybrid		Sowing(M/D)		V6(M/D)		R1(M/D)		R6(M/D)		Days before silking(d)		Effective accumulated temperature before silking(°C d)		Days after silking(d)		Effective accumulated temperatureafter silking (°C d)		Total growth period (d)		Total effective accumulated temperature (°C d)
2017	DH518		6/10		7/1		7/25		9/20		45		806.9		58		933.3		104		1740.2
	DH605		6/10		7/2		7/31		9/30		51		904.6		62		955.8		114		1860.4
2018	DH518		6/7		6/28		7/23		9/18		46		826.5		58		952.5		104		1779.0
	DH605		6/7		6/30		7/29		9/26		52		918.2		60		940.3		111		1858.4
2019	DH518		6/16		7/7		8/1		10/3		46		846.1		63		860.3		109		1647.1
	DH605		6/16		7/9		8/6		10/10		51		931.5		65		807.2		116		1719.9
2020	DH518		6/9		7/1		7/25		9/23		46		708.3		60		923.7		106		1632.0
	DH605		6/9		7/3		7/30		9/30		51		785.6		62		915.9		113		1701.5
2021	DH518		6/8		6/30		7/24		9/21		46		794.8		59		864.9		105		1659.7
	DH605		6/8		7/1		7/30		9/29		52		895.6		61		853.7		113		1749.3

DH518, Denghai518; DH605, Denghai605.

### Growth process and demand characteristics of GDD

The growth period of DH518 was shorter than that of DH605, and different period was mainly from V6 to R1 stage. The growth period of DH518 was 7-10 days shorter than that of DH605 and the pre-silking GDD was significantly lower. The pre-silking days and GDD of DH605 were increased 5-6 days and 77.3-100.8°C than those of DH518. There was no significant difference in post-silking GDD among different hybrids (**Table 2**). At post-silking, the yield per GDD of DH605 (12.73 kg ha^-1^ (°C d)^-1^) was significantly higher than that of DH518 (11.87 kg ha^-1^ (°C d)^-1^).

### Dry matter accumulation

**Figure 2:**
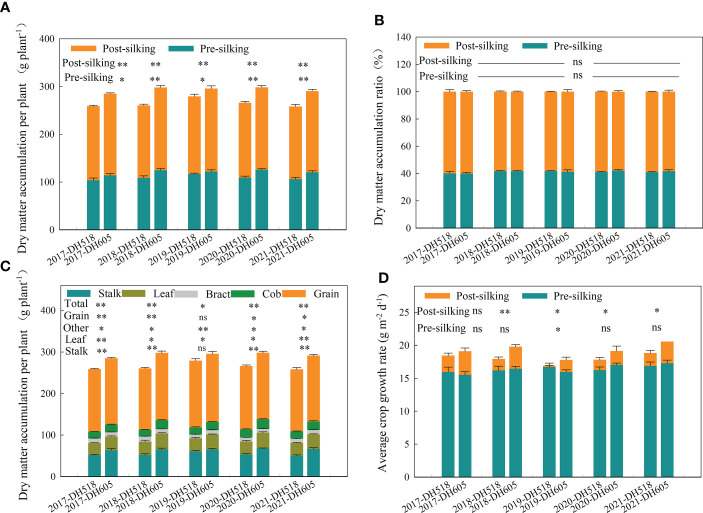
Above-ground biomass distribution and average crop growth rate of summer maize hybrids with different growth duration (2017-2021). 2017, 2018, 2019, 2020 and 2021 were different years; DH518, Denghai518; DH605, Denghai605. **(A)** Pre-silking and post-silking dry matter accumulation in aboveground biomass. Pre-silking and post-silking were the dry matter accumulation in aboveground biomass in that stage. **(B)** Pre-silking and post-silking dry matter accumulation ratio in aboveground biomass. Pre-silking and post-silking were the dry matter accumulation ratio` in aboveground biomass in that stage. **(C)** The dry matter distribution of different summer hybrids. Grain leaf and stalk were dry matter accumulation of each organ per plant; total were total dry matter accumulation per plant; other were dry matter accumulation of bract and cob per plant. **(D)** Average crop growth rate of different hybrids. Pre-silking and post-silking were average crop growth rate in that stage. ^**^ and ^*^ indicated significantly different at the 0.01 and 0.05 probability levels, respectively; ns means there is no significantly different at the 0.05 probability levels, respectively.

The PrSDMA and PoSDMA of DH605 were significantly higher than DH518. Compared with DH518, the PrSDMA and PoSDMA of DH605 increased by 9.7% and 10.2% respectively (**Figure 2A**). There was no significant difference between PrSDMAR and PoSDMAR of summer maize hybrids with different growth duration. The PoSDMA of the two hybrids was higher than the PrSDMA. Summer maize hybrids with different growth duration accumulated 40% of the total plant dry matter from sowing to R1 (**Figure 2B**), which indicated This shows that hybrids with higher PrSDMA had greater PoSDMA production potential. The DMA of various organs of DH605 was significantly higher than that of DH518. The DMA of grain, leaf, stalk and other organs of DH605 at R6 increased by 5.3-9.7%, 12.1-22.4%, 21.6-33.0% and 9.4-13.5%, respectively, compared to DH518. (**Figure 2C**)

### DMRE and DMRC of leaf or stalk

The DMR of leaf and DMRE of leaf and stalk of DH605 were significantly lower than those of DH518, but there was no significant difference in DMR of stalk between two hybrids. Compared with DH605, the DMRE of leaf and stalk of DH518 increased by 8.0-11.2% and 5.1-5.9%, respectively (**Table 3**). The DMRC of leaf or stalk of DH518 was significantly higher than that of DH605, and the contribution to grain dry matter by PoSDMA of different summer maize hybrids was 90.0-93.6%, which was significantly higher than DMRC. (**Table 4**).

**Table 3:** Dry matter remobilization of vegetative tissues of summer maize hybrids with different growth duration from 2017 to 2021.

Year	Hybrid	Dry matter remobilization of stalk (g)	Dry matter remobilization efficiency of stalk (%)	Dry matter remobilization of leaf (g)	Dry matter remobilization efficiency of leaf (%)
2017	DH518	17.81 ± 1.96a	25.49 ± 1.76a	5.26 ± 0.96a	15.38 ± 3.19a
	DH605	15.75 ± 1.32a	19.99 ± 1.98b	1.98 ± 1.17b	5.62 ± 3.35b
2018	DH518	19.36 ± 0.95a	27.20 ± 1.91a	5.36 ± 1.30a	14.21 ± 2.58a
	DH605	18.38 ± 2.00a	21.98 ± 2.42b	2.55 ± 0.87b	6.22 ± 2.18b
2019	DH518	19.68 ± 0.79a	24.65 ± 1.32a	5.19 ± 1.51a	14.07 ± 4.81a
	DH605	18.38 ± 0.49a	22.03 ± 0.72b	2.09 ± 0.42b	5.38 ± 0.94b
2020	DH518	19.16 ± 0.57a	26.51 ± 1.11a	6.01 ± 1.43a	16.13 ± 3.51a
	DH605	18.40 ± 0.67a	21.40 ± 0.66b	1.95 ± 0.42b	4.88 ± 1.12b
2021	DH518	18.82 ± 1.03a	27.34 ± 1.72a	5.87 ± 1.43a	15.82 ± 4.21a
	DH605	18.16 ± 0.46a	21.45 ± 1.03b	1.73 ± 0.47b	4.65 ± 1.13b
Year (Y)		0.989	0.570	0.131	0.062
Hybrid (H)		1.653	23.923^**^	68.011^**^	70.256^**^
Y × H		0.104	0.298	0.388	0.312

In the same year, means followed by a common letter are not significantly different by LSD test at the 5% level of significance. DH518, Denghai518; DH605, Denghai605. ** indicated significantly different at the 0.01 and 0.05 probability levels, respectively.

**Table 4:** Contribution to grain dry matter by leaf and stalk dry matter remobilization of summer maize hybrids with different growth duration (2017-2018; 2020-2021).

Year	Hybrid	DMR of leaf and stalk to grain(g)	DMRC of leaf and stalk(%)	Yield per GDD(kg ha^-1^°C^-1^ d^-1^)
2017	DH518	14.34 ± 2.02a	9.50 ± 1.27a	11.83 ± 0.14b
	DH605	10.53 ± 0.61b	6.61 ± 0.37b	12.49 ± 0.13a
2018	DH518	14.75 ± 0.22a	9.99 ± 0.02a	11.21 ± 0.22b
	DH605	13.29 ± 0.75b	8.20 ± 0.55b	12.42 ± 0.10a
2020	DH518	13.76 ± 1.01a	9.04 ± 0.59a	12.05 ± 0.21b
	DH605	10.32 ± 0.41b	6.43 ± 0.15b	13.29 ± 0.30a
2021	DH518	14.75 ± 0.48a	9.87 ± 0.05a	12.39 ± 0.40b
	DH605	11.03 ± 0.08b	6.95 ± 0.16b	13.90 ± 0.30a
Year (Y)		3.830^*^	4.147^*^	34.123^**^
Hybrid (H)		43.622^**^	70.621^**^	133.933^**^
Y × H		1.733	0.975	3.177

DMR, dry matter remobilization; DMRC, contribution to grain dry matter by leaf and stalk dry matter remobilization. In the same year, means followed by a common letter are not significantly different by LSD test at the 5% level of significance. DH518, Denghai518; DH605, Denghai605. ** and * indicated significantly different at the 0.01 and 0.05 probability levels, respectively.

**Figure 3:**
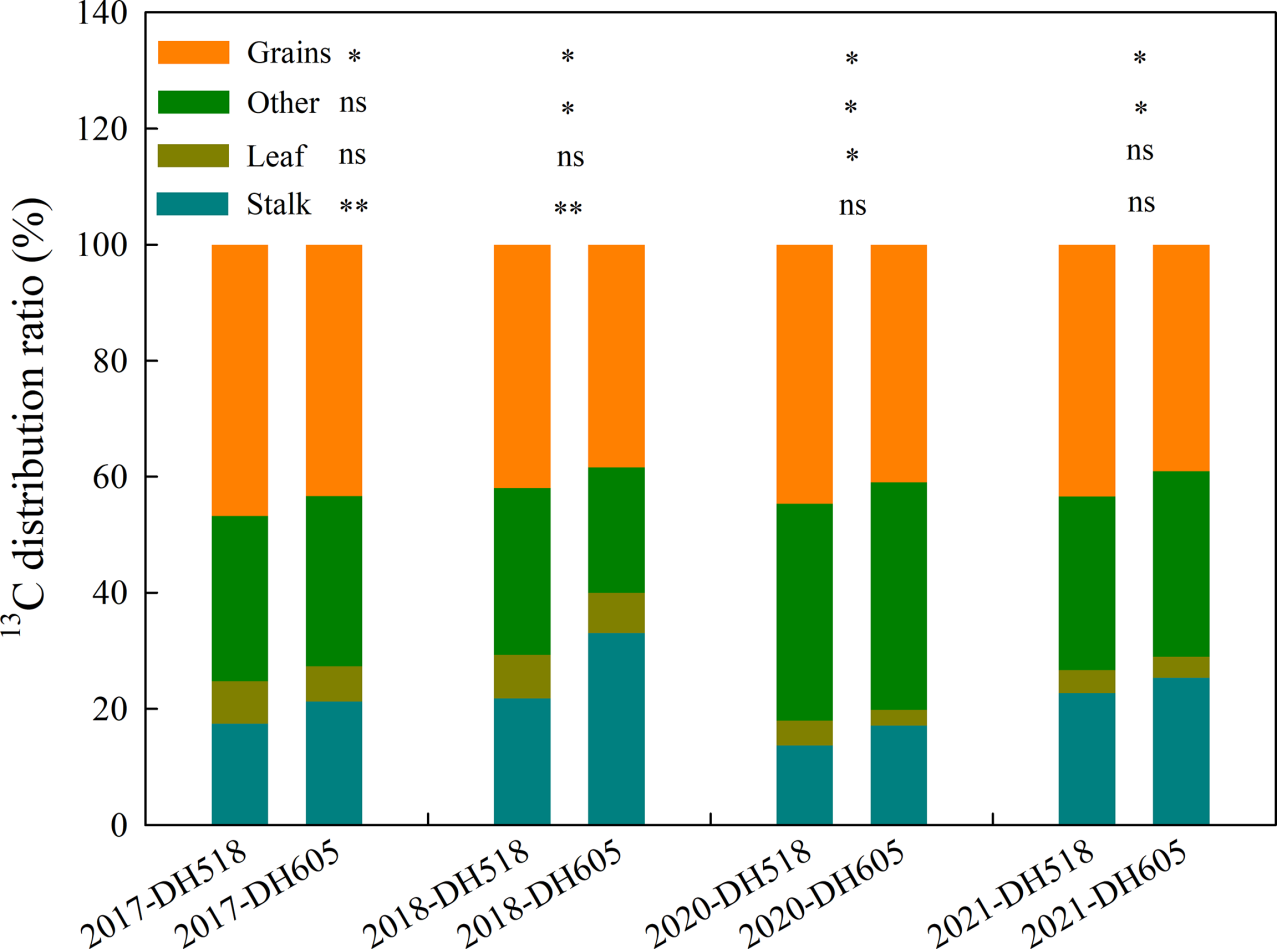
^13^C distribution to organs of summer maize hybrids with different growth duration (2017-2018; 2020-2021). 2017, 2018, 2019, 2020 and 2021 were different years; DH518, Denghai518; DH605, Denghai605. Grain, leaf and stalk were ^13^C distribution ratio to each organ; other were ^13^C distribution ratio of bract and cob. ^**^ and ^*^ indicated significantly different at the 0.01 and 0.05 probability levels, respectively; ns means there is no significantly different at the 0.05 probability levels, respectively.

Compared with DH605, the ^13^C distribution to grains of DH518 increased by 3.4-4.4%, while the ^13^C distribution in stalks decreased by 2.6-11.2%. The distribution of ^13^C in different organs of different summer maize hybrids is as follows: grain>others>stalk>leaf. The proportion of ^13^C in ear is more than 60% (**Figure 3**).

### Photosynthetic characteristics

**Figure 4:**
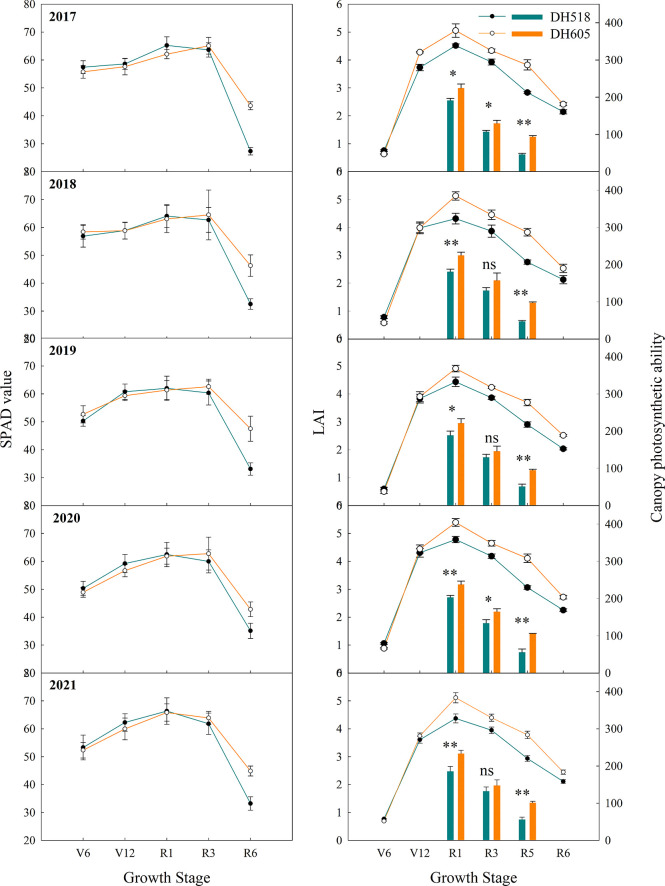
Chlorophyll relative content, leaf area index and canopy photosynthesis ability of summer maize hybrids with different growth duration (2017-2021). 2017, 2018, 2019, 2020 and 2021 were different years; DH518, Denghai518; DH605, Denghai605. V6, V12, R1, R3 and R6 were the sixth leaf stage, the 12th leaf stage, silking stage, milk stage and physiological maturity stage, respectively. ^**^ and ^*^ indicated significantly different at the 0.01 and 0.05 probability levels, respectively. ns means there is no significantly different at the 0.05 probability levels.

The dynamic variation in the post-silking population leaf area was shown in **Figure 4**. The overall change trend of the LAI of DH518 and DH605 were the same, which gradually increased from V6-R1 stage, at R1 the LAI was the highest and gradually decreased after R1 (**Figure 4**). The LAI of DH605 at the R1 and post-silking was significantly higher than that of DH518. Compared with DH518, the LAI of DH605 in R1, R3 and R6 increased by 10.8-19.0%, 9.6-15.1% and 12.7-23.6%, respectively. The change of post-silking LAI conformed to the Gaussian model. The analysis showed that the LAI_max_ of the two hybrids appeared 3-8 days after silking. The DLAI of DH605 was more than 56 days, while that of DH518 was less than 51 days, which significantly accelerated the rapid decay process of DH518. Canopy photosynthetic capacity is a comprehensive reflection of *P_n_* and LAI. The post-silking canopy photosynthetic capacity of DH605 was significantly higher than that of DH518. Compared with DH518, the canopy photosynthetic capacity of DH605 in R1, R3 and R5 increased by 17.0-25.9%, 11.5-22.8% and 79.5-104.3%, respectively. The SPAD value of DH605 was significantly higher than that of DH518 at R6, and the SPAD value of DH605 increased by 21.9-59.7% compared with DH518 at R6. There was no significant difference between different hybrids before R3 (**Figure 4**).

**Figure 5:**
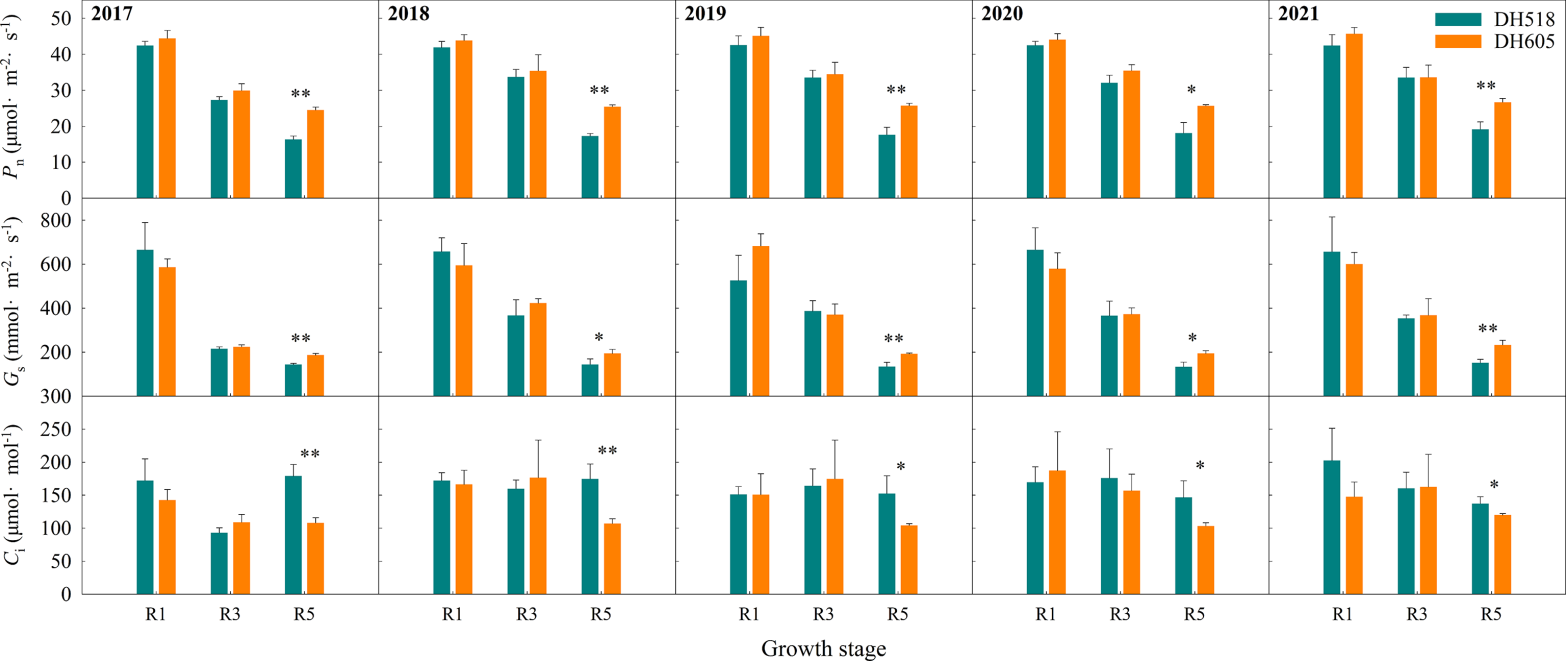
Post-silking changes of gas exchange parameters in leaves of summer maize hybrids with different growth duration (2017-2021). 2017, 2018, 2019, 2020 and 2021 were different years; DH518, Denghai518; DH605, Denghai605. R1, R3 and R5 were silking stage, milk stage and 45 days after silking. *P_n_*, the net photosynthetic rates; *G_s_*, stomatal conductance; *C_i_*, intercellular CO_2_ concentration. ^**^ and ^*^ indicated significantly different at the 0.01 and 0.05 probability levels, respectively.

The *P_n_*, *G_s_* and *C_i_* of ear leaf of summer maize hybrids with different growth duration were not significantly different at R1 and R3 stages (**Figure 5**). The *P_n_* and *G_s_* of DH605 was significantly higher than those of DH518, and the *C_i_* of DH605 was significantly lower than that of DH518. Compared with DH605, the *P_n_* and *G_s_* of DH518 decreased by 28.2-33.3% and 22.7-35.1%, respectively, and the *C_i_* increased by 14.4-65.7%, indicating that the decrease of *P_n_* in the later stage of DH518 was limited by stomatal factors and the decline of mesophyll cell function. The linear fitting of *P_n_* after full expansion of ear leaf showed that the APD of DH605 was more than 10 days longer than that of DH518 (**Table 5**).

**Table 5:** Active photosynthesis duration of summer maize hybrids with different growth duration from 2017 to 2021.

Year	Hybrid	Linear fitting equation	Correlation coefficient	*Pi*	APD
2017	DH518	y=43.34-0.58x	0.994	46.07	40.27
	DH605	y=45.04-0.47x	0.989	48.23	50.67
2018	DH518	y=43.97-0.52x	0.946	46.33	44.34
	DH605	y=45.40-0.41x	0.958	48.13	57.02
2019	DH518	y=44.94-0.54x	0.952	47.83	43.11
	DH605	y=45.93-0.43x	0.973	48.37	55.76
2020	DH518	y=45.07-0.55x	0.967	48.57	41.79
	DH605	y=46.04-0.42x	0.966	49.27	55.57
2021	DH518	y=44.75-0.51x	0.952	47.63	45.09
	DH605	y=46.19-0.43x	0.983	48.70	55.84

Pi, initial net photosynthetic rate; APD, active photosynthesis duration (the days from Pi to half of Pi).

### The correlation analysis

Correlation analyses showed different relationships between yield and DMA and HI of summer maize hybrids with different growth duration. The yield of DH518 was positively correlated with dry matter and harvest index. DH518 reached significant levels in 2017 and 2020, and DH605 reached significant levels in 2018,2020 and 2021 (**Table 6**). The HI was negatively correlated with the days from sowing to R1 (r=0.922, *p*<0.01), but not with the days of R1-R6 (*p*>0.05) (**Figure S1**).

**Table 6:** Regression analysis between grain yield and DMA and HI of summer maize hybrids with different growth duration from 2017 to 2021.

Year	Hybrid	Yield × DMA (y= a + bx)			Yield ×HI (y= a + bx)		
		a	b	r	a	b	r
2017	DH518	43.544	0.018	0.999*	0.554	2.27×10^-06^	0.633
	DH605	-64.458	0.027	0.995	-0.231	6.18×10^-05^	0.999*
2018	DH518	-43.551	0.026	0.919	0.294	2.30×10^-05^	0.912
	DH605	-90.840	0.031	0.999*	0.393	1.19×10^-05^	0.845
2019	DH518	-92.757	0.030	0.981	0.159	3.40×10^-05^	0.762
	DH605	-50.962	0.027	0.994	-0.361	7.20×10^-05^	0.947
2020	DH518	-57.909	0.026	1.000**	0.426	1.20×10^-05^	0.893
	DH605	81.237	0.017	1.000*	0.339	1.52×10^-05^	0.938
2021	DH518	64.431	0.016	0.937	0.465	9.55×10^-06^	0.996
	DH605	-29.644	0.025	0.998*	0.157	3.04×10^-05^	0.943

r, correlation coefficient. ^**^ and ^*^ indicated significantly different at the 0.01 and 0.05 probability levels, respectively. DH518, Denghai518; DH605, Denghai605.

## Discussion

### The light and temperature resource use efficiency from silking to physiological maturity stage of different summer maize hybrids

In the past few decades, breeding programs in pursuit of higher yields have extended the fertility period of maize (Valentinuz and Tollenaar, 2004; Luque et al., 2006). Compared with early maturity hybrids, the senescence of mid-late maturity hybrids occurs slower (Valentinuz and Tollenaar, 2004), which shows that the post-silking chlorophyll activity steady phase (RSP) of mid-late maturity hybrids lasted longer (Ding et al., 2005). Our results showed that the decline rate of SPAD value of DH605 was lower than that of DH518 after silking, which greatly delayed the senescence of leaves (**Figure 4**). Late maturity hybrids have higher leaf area index, longer photosynthesis time, slower leaf senescence and higher DMA rate at filling stage (Ma and Dwyer, 1998; Borrell et al., 2001; Duvick, 2005), which has a positive effect on the increase of yield (Rajcan and Tollenaar, 1999; Valentinuz and Tollenaar, 2004; Ding et al., 2005). The photosynthetic characteristics of leaves are closely related to the yield (Motto, 1987; Ma and Dwyer, 1998). DH605 had larger leaf area than that of DH518. The time of DH605 entering the rapid decay stage was more than 15 days later than that of DH518 (**Figure 4**). The DLAI of DH605 was 5 days and the APD of DH605 was more than 10 days longer than that of DH518 (**Table 7**). There is no doubt that the yield of mid-late maturity hybrids is higher than that of early maturity hybrids (Scott and Hector, 1997; Morinaka et al., 2006; **Table 1**), not only because of the extension of growth period (**Table 2**), but also because DH605 has higher light and temperature resource use efficiency. When the GDD was increased by 1 °C, the yield of DH605 was higher than that of DH518 by 0.86kg ha^-1^. Therefore, the light and temperature resource use efficiency from silking to physiological maturity stage is key to determine the yield of summer maize hybrids with different growth duration. In the future, in the research on the yield of summer maize hybrids with different growth duration, we should pay attention not only to the changes of growth period of different hybrids, but also to the differences of light and temperature resource use efficiency from silking to physiological maturity stage. In addition, it is necessary to further explore the physiological and molecular mechanisms that cause this difference, such as changes in the expression of genes and metabolic enzymes related to plant senescence.

**Table 7:** Characteristic parameters of LAI fixt by Gaussian Model of summer maize hybrids with different growth duration from 2017 to 2021.

Year	Hybrid	A	B	C	R	DLAI
2017	DH518	4.56	5.41	42.62	0.996	50.09
	DH605	5.07	5.65	48.34	0.992	56.95
2018	DH518	4.40	7.96	40.45	0.995	47.64
	DH605	5.15	5.72	48.03	0.996	56.48
2019	DH518	4.48	6.28	42.31	0.999	49.92
	DH605	4.91	3.28	52.39	0.996	61.72
2020	DH518	4.83	5.75	43.10	0.997	50.75
	DH605	5.39	4.73	50.88	0.993	59.87
2021	DH518	4.45	7.66	42.89	0.997	50.34
	DH605	5.12	4.97	48.60	0.996	57.23

A, LAI_max_; B, the days of LAI_max_ occurred; C, kurtosis of Gaussian curve; R, determinate coefficient; DLAI, the duration of higher LAI (the days from LAI_max_ to half of LAI_max_).

### Accumulation, distribution and transport of dry matter in different summer maize hybrids

Grain filling of cereal crops depends on two sourcesof photosynthate, current photosynthate transferred directly to the grain and redistribution of photosynthate from reserve pools in vegetative tissues (Johnson and Tanner, 1972; Pheloung and Siddique, 1991; Kobata et al., 1992; Schnyder, 1993; Blum et al., 1994; Yang and Zhang, 2006). The characteristics of PrSDMR and PrSDMRC of different summer maize hybrids have been widely studied (Ning et al., 2013; Horacio et al., 2014; Chen et al., 2015a). However, the calculation of the proportion of DMRE and pre-silking and post-silking DMRC are not accurate. For example, about 40% of the PrSDMA was used to meet the development of other organs such as cob and bract (**Figure 3**), which was not taken into account in most articles. Therefore,^13^C pulse labeling technique was used to investigate the dry matter distribution characteristics of summer maize hybrids with different growth duration in this experiment. The ^13^C distribution to grains of DH518 was significantly higher than that of DH605, and the contribution rate to yield of HI and the HI of DH518 was significantly higher than that of DH605 (**Table 8**; **Figure S2**). This may be related to the activities of related enzymes in the process of sucrose transport, and higher amylase and SPS activities can promote starch degradation and photosynthetic redistributed from stalk to grain (Wang and Zhang, 2020). The next step could be to focus on the changes in the activities of these related enzymes to explain the reasons for the higher HI of DH518. This indicates that DH518 distributes more photosynthate from stalks to grains to ensure grain development. Non-structural carbohydrates stored in vegetative tissues can be remobilized and exported to grains during grain filling (Yang et al., 2001), but their contribution is limited (Cliquet et al., 1990). In wheat, 7-36% of the yield comes from the PrSDMR, and most of the yield comes from current photosynthate transferred directly (Austin et al., 1977; Papakosta and Gagianas, 1991; Masoni et al., 2007). The duration of sowing-R1 were 5-6 days shorter than that of DH605 (**Table 1**). The results based on ^13^C pulse labeling showed that the DMRC of DH518 was significantly higher than that of DH605 (**Table 4**)., which indicated that the shortening of sowing-R1 had no negative effect on yield. Most of the grain dry matter of maize comes from the photosynthetic products produced during grain filling (Tollenaar et al., 2004; Lee and Tollenaar, 2007), which is supported by the post-silking slight decrease of dry matter in vegetative tissues (**Table 3**). The PoSDMA of DH605 was significantly higher than that of DH518 (**Figure 2**). More PoSDMA may eventually make a significant contribution to higher yield. Our results showed that the contribution rate of PoSDMA to the yield of different summer maize hybrids was more than 90% (**Table 4**), which played a decisive role in the increase of yield.

**Table 8:** Grain/leaf and harvest index of summer maize hybrids with different growth duration from 2017 to 2021.

Year	Hybrid	Grain/leaf(kg m^-2^)	Harvest index
2017	DH518	0.268 ± 0.003a	0.582a
	DH605	0.252 ± 0.003b	0.559b
2018	DH518	0.269 ± 0.005a	0.567a
	DH605	0.247 ± 0.002b	0.544b
2019	DH518	0.274 ± 0.004a	0.576a
	DH605	0.259 ± 0.003b	0.554a
2020	DH518	0.253 ± 0.004a	0.572a
	DH605	0.241 ± 0.005b	0.537b
2021	DH518	0.267 ± 0.009a	0.578a
	DH605	0.249 ± 0.005b	0.544b
Year (Y)		12.697^**^	6.399^**^
Hybrid (H)		91.963^**^	134.497^**^
Y × H		0.990	1.491

In the same year, means followed by a common letter are not significantly different by LSD test at the 5% level of significance. DH518, Denghai518; DH605, Denghai605. ** indicated significantly different at the 0.01 and 0.05 probability levels, respectively.

### HI and DMA of summer maize hybrids with different growth duration

**Figure 6:**
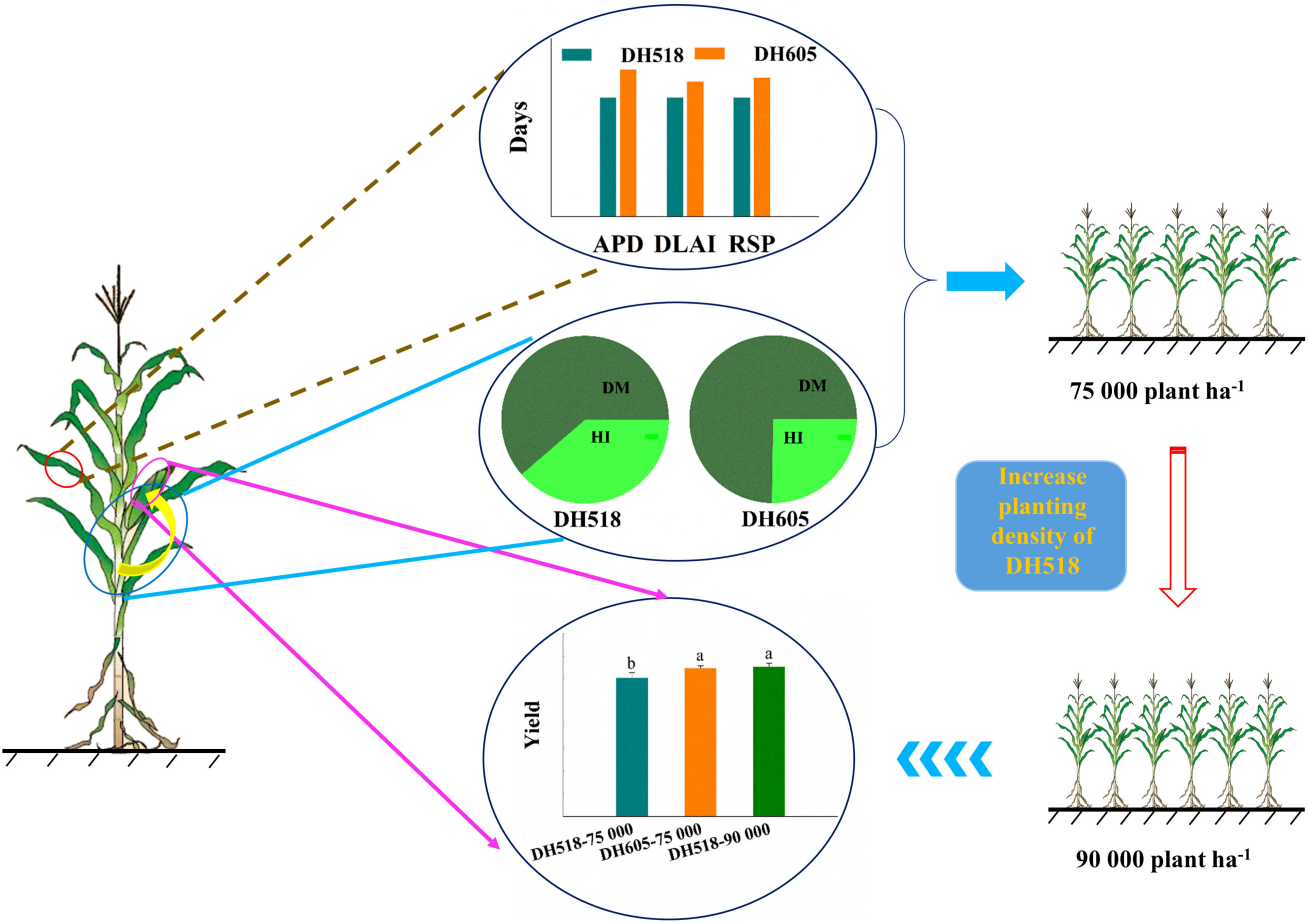
Grain yield, photosynthetic characteristics and contribution of dry matter accumulation and harvest index to yield of summer maize hybrids with different growth duration. The shortening of active photosynthesis duration, the duration of higher LAI and chlorophyll relative steady phase of early-maturing hybrids reduced the post-silking utilization efficiency of light and temperature resources and negatively affected the yield. Increasing planting density of early-maturing hybrids and increasing dry matter accumulation of population could significantly increase yield, and there was no significant difference between early-maturing hybrids and middle-late maturing hybrids. DH518, Denghai518; DH605, Denghai605. ADP, active photosynthesis duration; DLAI, the duration of higher LAI; RSP, chlorophyll relative steady phase. Different letters on bars indicate significant differences among treatments at *p*< 0.05 using LSD test.

The effects of HI and DMA on yield varied with crop species. The wheat yield was mainly attributed to the increase in HI (Nass, 1980; Brancourt-Hulmel et al., 2003; Xiao et al., 2012; Lo Valvo et al., 2017). In rice, the increase in yield before 1980 was related to the increase in HI, while after 1980 that was due to the increase in biomass production (Peng et al., 2000), which was similar to the succession of Chinese Japonica hybrids (Xiong et al., 2011). So far, hybrid rice and super rice have higher yields, which was mainly due to an increase in DMA rather than an increase in HI (Yang et al., 2006). However, maize as a C_4_ plant is different from most small-grain crops. Our results showed that the increase in DH518 yield in different years was mainly due to the increase in DMA and HI, while the increase in the yield of DH605 in different years was mainly depended on the increase in DMA. The grain/leaf ratio and HI of the population could be a better way to reflect the source-sink relationship of the population (Rajcan and Tollenaar, 1999). According to the theoretical value of the target yield of 15,000kg ha^-1^ and the LAI was 5-6 when intercepting 95% solar radiation, the grain-leaf ratio was 0.3-0.25 (Wang, 2008). The grain/leaf ratio of different summer maize hybrids was higher than 0.25 kg·m^-2^ (**Table 8**). On the premise of increasing the grain-leaf ratio, increasing the density and “expanding the pool and strengthening the source” can make the leaves per unit area to supply more grains and further increase the yield. With the increase of density, the DMA of maize per unit area increased significantly (Duvick, 2005; Li et al., 2015). Our results showed that the contribution rate of DMA to yield of different summer maize hybrids was higher than that of HI (**Figure S2**). Our previous studies have confirmed that the yield of DH518 was significantly lower than that of DH605 under 75,000 ha^-1^ planting density. There was no significant difference between the yield of DH518 at 90,000 plants ha^-1^ and that of DH605 at 75,000 plants ha^-1^, both of the two treatment yields were significantly higher than that of DH518 under 75,000 ha^-1^ planting density. (Wan et al., 2018; **Figure 6** and **S3**). Therefore, the yield loss caused by the decrease of the light and temperature resource use efficiency from silking to physiological maturity stage could be made up by increasing the planting density of DH518 to increase the population DMA and light and temperature resource use efficiency.

## Conclusion

The yield of DH605 was significantly higher than that of DH518. The difference in the growth period of different hybrids was mainly in the V6 to R1 stage. Compared to DH518, the higher light and temperature resource use efficiency from silking to physiological maturity stage of DH605 led to higher yield. The shortening of the growth stage of DH518 from sowing to R1 stage could make up for the yield loss by increasing the harvest index. The yield of DH518 could be increased by reasonably increasing planting density. Therefore, it is necessary to further study the carbon transport process of the plant to explain the association between the shortened growth period and the increased HI of early maturity hybrid. This study can provide a feasible research direction for the breeding and high yield and high efficiency cultivation of early maturity summer maize in the future.

## Data availability statement

The original contributions presented in the study are included in the article/**Supplementary Material**. Further inquiries can be directed to the corresponding author.

## Author contributions

JyZ: Data curation, writing—original draft, visualization, and investigation. BR, BZ, and PL: Supervision. JwZ: Conceptualization, writing—review and editing, and funding acquisition. All authors contributed to the article and approved the submitted version.

## Funding

This work was supported by Province Key Research and Development Program of Shandong (2021LZGC014-2), National Natural Science Foundation of China (32172115), the earmarked fund for CARS (CARS-02-21).

## Conflict of interest

The authors declare that the research was conducted in the absence of any commercial or financial relationships that could be construed as a potential conflict of interest.

## Publisher’s note

All claims expressed in this article are solely those of the authors and do not necessarily represent those of their affiliated organizations, or those of the publisher, the editors and the reviewers. Any product that may be evaluated in this article, or claim that may be made by its manufacturer, is not guaranteed or endorsed by the publisher.

## Supplementary material

Supplementary Figure 1 Relationships between days at different growth duration and HI of summer maize (2017-2021). HI, harvest index. R1, silking stage; R6, physiological maturity stage. ^**^ indicated significantly different at the 0.01 probability levels, ns means not significant at the level of *p*< 0.05.

Supplementary Figure 2 Contribution rates of DM and HI to grain yield (2017-2021). DH518, Denghai518; DH605, Denghai605. DMA, dry matter accumulation; HI, harvest index.

Supplementary Figure 3 Yield of summer maize hybrids with different growth duration (2016-2017). DH518, Denghai518; DH605, Denghai605. 75 000 and 90 000 were the planting density. Different letters on bars indicate significant differences among treatments at *p*< 0.05 using LSD test.
