# Supplementary figures and images for: Yield of summer maize hybrids with different growth duration determined by light and temperature resource use efficiency from silking to physiological maturity stage
Source: Front Plant Sci. 2022 Sep 29;13:992311. doi: 10.3389/fpls.2022.992311 (PMC9557109; doi:10.3389/fpls.2022.992311)

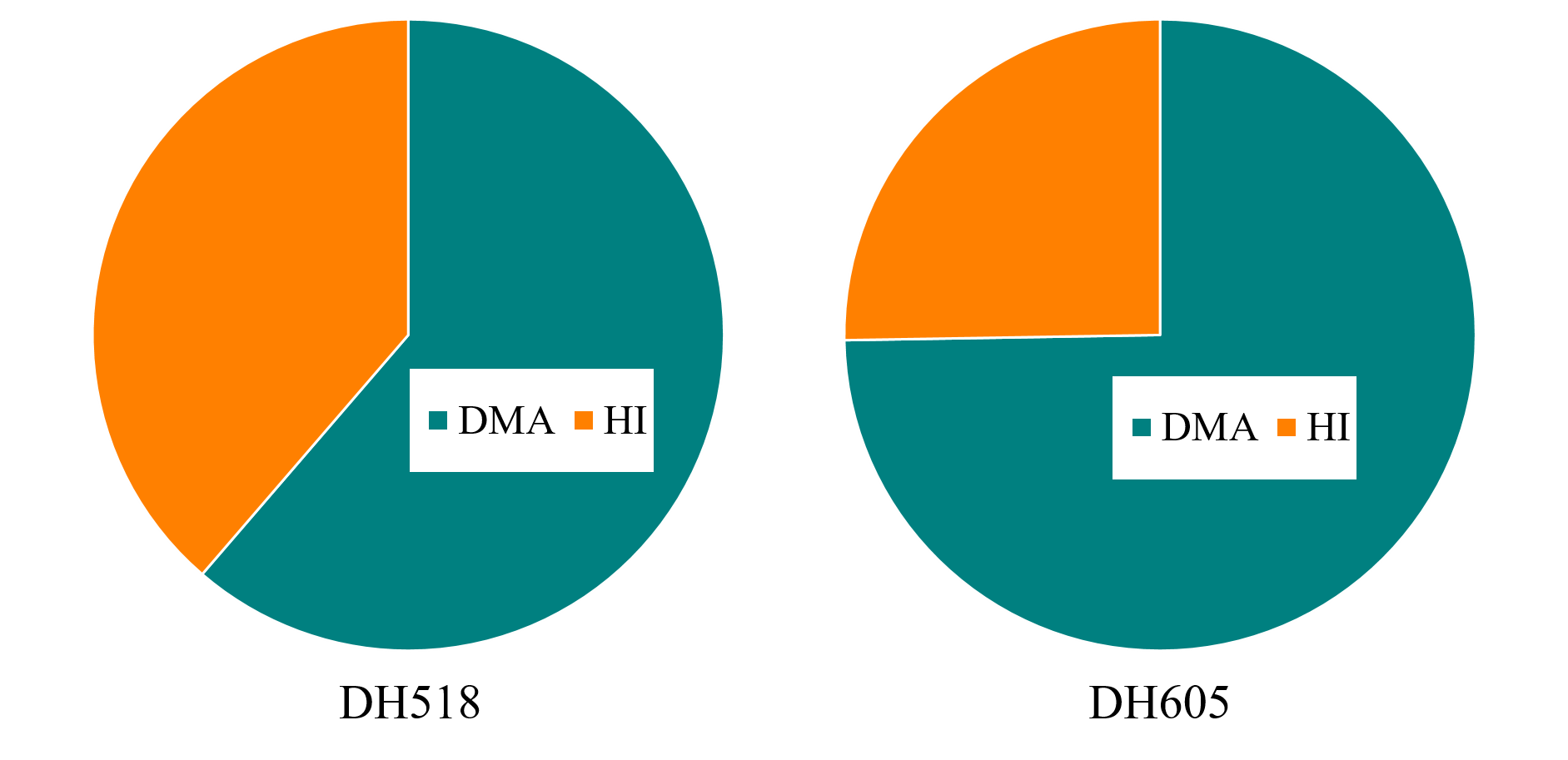

Supplement: Supplementary Figure 2 — Contribution rates of DM and HI to grain yield (2017-2021). DH518, Denghai518; DH605, Denghai605. DMA, dry matter accumulation; HI, harvest index. [file Image_2.tif]
